# VEGF Pathway Gene Expression Profile of Proliferating versus Involuting Infantile Hemangiomas: Preliminary Evidence and Review of the Literature

**DOI:** 10.3390/children9060908

**Published:** 2022-06-17

**Authors:** Rodica Elena Heredea, Eugen Melnic, Laura Elena Cirligeriu, Patricia Lorena Berzava, Maria Corina Stănciulescu, Călin Marius Popoiu, Anca Maria Cimpean

**Affiliations:** 1 Center of Expertise for Rare Vascular Disease in Children, Louis Turcanu Children Hospital, 300041 Timisoara, Romania; elena-rodica.heredea@umft.ro (R.E.H.); mcpopoiu@umft.ro (C.M.P.); acimpeanu@umft.ro (A.M.C.); 2 Department V/Division of Clinical Practical Skills, Victor Babes University of Medicine and Pharmacy, 300041 Timisoara, Romania; 3 Department of Pathology, State University “Nicolae Testemitanu” University of Medicine and Pharmacy, 2004 Chisinau, Moldova; eugenmelnic@gmail.com; 4 3rd Department, Discipline of Restorative Dentistry and Endodontics, ‘Victor Babes’ University of Medicine and Pharmacy, 300070 Timisoara, Romania; 5 Research Center TADERP, Faculty of Dental Medicine, ‘Victor Babes’ University of Medicine and Pharmacy, 300070 Timisoara, Romania; 6 Department of Microscopic Morphology/Histology, ‘Victor Babes’ University of Medicine and Pharmacy, 300041 Timisoara, Romania; patricia.berzava@yahoo.com; 7 Angiogenesis Research Center Timisoara, ‘Victor Babes’ University of Medicine and Pharmacy, 300041 Timisoara, Romania; 8 Department XI/Pediatric Surgery, ‘Victor Babes’ University of Medicine and Pharmacy, 300041 Timisoara, Romania

**Keywords:** VEGF pathway, involuting hemangioma, proliferating hemangioma, gene expression profile

## Abstract

*Background*. Infantile hemangiomas may have unexpected behavior. Initial regression (spontaneously or drug-induced) may be followed by unexplained recurrences. At this moment, there are no well-established criteria to predict infantile hemangioma reccurrences. *Methods*. We compared the VEGF pathway gene expression profile for one case of involuting infantile hemangioma versus one case of recurrent proliferative infantile hemangioma using TaqMan Array. *Results*. We found ten genes upregulated for both involuting and recurrent proliferative hemangiomas: ACTB, KRAS, MAP2K1, HRAS, NOS3, BAD, HSPB1, HPRT1, GUSB, and CASP9. Thirteen genes were downregulated for both involuting and proliferative hemangiomas: FIGF, ACTG1, GRB2, MAPKAPK2, ACTG2, MAP2K2, MAPK3, HSP90AA1, MAP2K6, NRAS, ACTA1, KDR, and MAPK1. Three genes showed divergent expression between proliferating and involuting hemangiomas. Proliferating hemangioma had MAPK14 and AKT1 gene upregulation and ACTA2 downregulation. Involuting infantile hemangioma was characterized by ACTA2 upregulation and AKT1 and MAPK14 downregulation. *Conclusions*. Three genes, AKT1, p38/MAPK14, and ACTA2, were found to have divergent expression in proliferating and involuting infantile hemangiomas. Excepting AKT1, which was mentioned in the last ISSVA classification (strictly related to Proteus Syndrome), none of the other genes were reported. An accurate gene expression profile mapping of infantile hemangiomas together with a gene expression-based hemangioma classification is stringently needed.

## 1. Introduction

Infantile hemangiomas are the most common benign vascular tumors of infancy and usually regress spontaneously [1]. In some cases, this regression does not appear and proliferative status persists, and sometimes these cases are refractory to therapy [2].

Propranolol therapy is widely used for treating infantile hemangiomas, but with variable results and, sometimes, with the occurrence of post-treatment recurrences. The recurrence rate of infantile hemangioma after propranolol therapy remains quite high [3], and the mechanism of action of propranolol in hemangiomas is not fully elucidated [4]. In vitro, recent data suggest that propranolol treatment interferes with the VEGF-mediated pathway and induces the inhibition of VEGF-mediated human umbilical vein endothelial cells (HUVECs) proliferation, migration, and tube formation, all well-known steps of the angiogenic process [5]. The hypothesis of excessive angiogenesis as the main pathogenic mechanism of hemangioma development is increasingly mentioned [6]. Angiogenic growth factors in infantile hemangiomas are currently being extensively studied, most likely due to an increased need for the development of novel therapies that may reduce recurrence rates and have few-to-no side effects. Recently, several papers reported that VEGF and bFGF serum levels decreased following propranolol therapy [6,7,8]. Moreover, some studies found VEGF as a negative prognostic factor involved in proliferative infantile hemangiomas, compared to involuting hemangiomas [9]. Although the serum level of VEGF and other growth factors has been extensively studied [9,10], VEGF pathway gene expression profile differences between proliferating and involuting hemangiomas are yet to be elucidated. Besides its role in beta-blocking, propranolol may regulate cell proliferation in hemangiomas via catecholamines and the VEGF pathway [11]. Furthermore, the intrinsic causes of recurrence in the case of hemangiomas treated with propranolol are not well known. VEGF overexpression in the proliferative phases of infantile hemangiomas is widely accepted, but the existence of biomarkers for assessing the therapeutic efficiency and for predicting post-therapy recurrence has not been outlined thus far. Several recent papers reported methods for assessing VEGF levels as biomarkers for infantile hemangiomas, but none of them have been validated for clinical use [12,13].

Based on the details described above, we hypothesize that the VEGF pathway gene expression profile may influence infantile hemangiomas through involuting or proliferative behavior and may induce resistance to therapy.

We present here a comparative assessment of the VEGF pathway gene expression profile of one case of involuting infantile hemangioma versus one case of recurrent proliferative infantile hemangioma before therapy. Both were subsequently treated with propranolol, followed by complete regression of involuting infantile hemangioma and a recurrence of proliferative hemangioma 9 months after the completion of propranolol therapy.

## 2. Materials and Methods

*Patients and biopsies*. Two facial skin hemangiomas biopsies from children aged 6 and 9 months were harvested for histopathologic diagnosis and genic analysis. The biopsies were harvested before applying any therapy and after obtaining informed consent from the children’s parents who agreed to use the tissues for molecular analysis. One of the patients was diagnosed with involuting infantile hemangioma, while the other was diagnosed with proliferating infantile hemangioma. Propranolol therapy was started for a period of 9 months at a dose of 3 mg/kg/day. Nine months later, patients came for routine follow-up. Involuting infantile hemangioma had a favorable evolution, while proliferative hemangioma recurred.

*TaqMan Assay. TaqMan Array Human VEGF Pathway* was used for accurate assessment of an entire gene signature in one simple experiment. The 96-well pre-configured plates for the TaqMan^®^ Gene Expression Assay were used for the VEGF pathways. The TaqMan^®^ Array Human VEGF Pathway 96-well plate contains 44 assays to VEGF Pathway associated genes and 4 assays to endogenous control genes. All assays are performed in triplicate.

**Figure 1:**
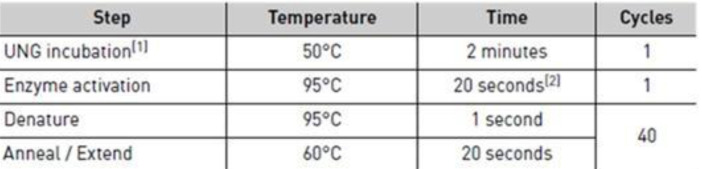
The TaqMan Array working protocol applied to hemangioma samples for VEGF pathway gene expression profile assessement.

*RNA extraction and cDNA synthesis*. The total RNA purification was performed using magnetic particle-based technology according to a standardized protocol for RNA extraction from fresh tissues, supplied with a Thermo Scientific™ KingFisher™ Pure RNA Tissue Kit (code 98040196/98040496. Thermo Scientific, Waltham, MA, USA) We established the RNA extraction by using 20 mg of hemangioma tissue which was mechanically disrupted in the lysis buffer, then, we loaded the material into an Automated King Fisher Duo Prime purification system (Thermo Scientific, Santa Clara, CA, USA). The total amount of RNA was checked using a Qubit™ 3.0 fluorometer (Thermo Scientific, Santa Clara, CA, USA). by preparing the preliminary samples with the Qubit™ RNA HS Analysis Kit (code Q32852). cDNA was obtained from the extracted RNA using a high-capacity RNA-to-cDNA kit™ (Applied Biosystems) following the protocol provided by the manufacturer. The TaqMan Array plates were prepared by loading 20 microliters of cDNA sample prepared using 10 μL of nuclease-free water and 10 μL of Master Mix (2X) on each well. The TaqMan Array Human VEGF plates were subjected to the RT-PCR technique performed on the 7500 Fast Dx Real-Time PCR Instrument (Applied Biosystem, USA), following the protocol described in Figure 1.

TaqMan assay results were evaluated using Data Assist V.3.01 software. A heatmap was automatically generated from this analysis. 

## 3. Results

**Figure 2:**
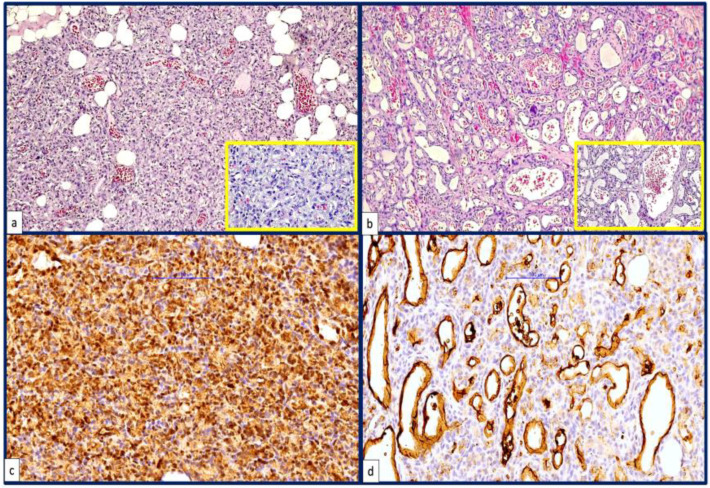
Histopathology and immunohistochemistry of proliferating versus involuting hemangioma. (**a**) Microscopically, a proliferating hemangioma is defined by the proliferation of vascular structures with lobular arrangement, with the lobules delineated by fibrous septa. The vascular structures have a predominantly small caliber, forming solid masses of proliferating cells. (**b**). Involuting hemangioma showing proliferation of vascular structures of variable caliber, some of them highly dilated and lined with flattened endothelial cells. Bands of fibrosis are identified as dissecting tumor proliferation. (**c**,**d**) Both infantile hemangiomas expressed GLUT1.

Histopathologic evaluation of two infantile hemangiomas confirmed that one case had microscopic features of a proliferating hemangioma (Figure 2a) and the other was an involuting hemangioma (Figure 2b). Both were positive for GLUT1 (Figure 2c,d).

**Figure 3:**
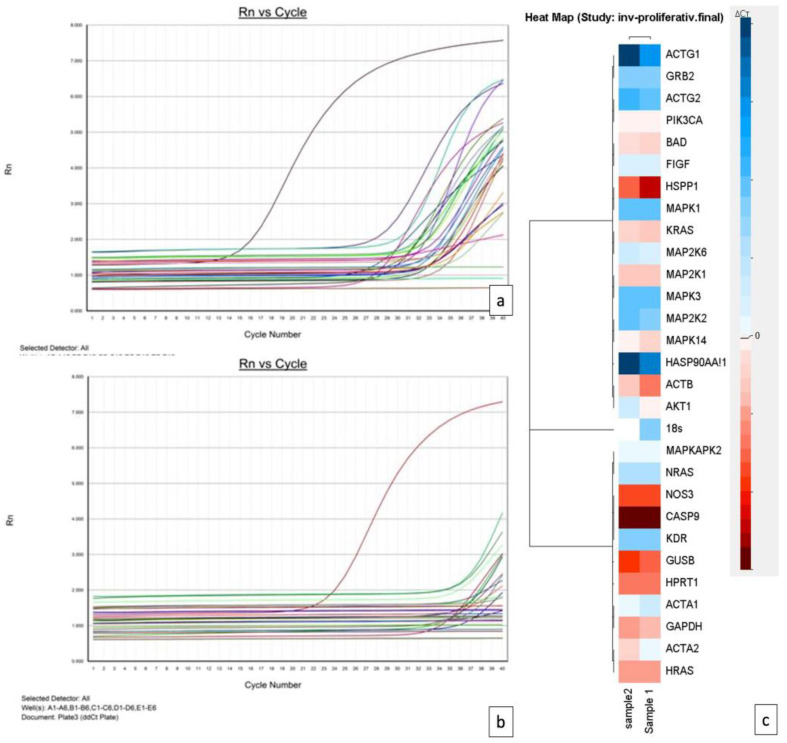
Amplification plots from proliferating (**a**) and involuting (**b**) hemangiomas and a comparative heatmap (**c**) generated using Data Assist software based on RT PCR VEGF pathway gene expression profile assessment.

TaqMan analysis of the VEGF pathway gene expression profile revealed a heterogeneity of gene expression between involuting and proliferating hemangiomas. Twenty-six genes of the VEGF pathway were considered in the present study. VEGF-A and VEGF-C genes were upregulated for both types of hemangiomas. The ddCt comparative assessment of the VEGF pathway gene expression profile in proliferative hemangiomas and involuting hemangiomas is shown using amplification plots (raw data) presented in Figure 3a,b. Based on analysis of VEGF gene expression profile parameters, a heatmap was automatically generated using Data Assist software. (Figure 3c).

Three genes had divergent expression between involuting and proliferating hemangiomas.

The most prominent divergent expression was found for the Alpha-actin-2 gene (ACTA2), which was downregulated in proliferating hemangioma and upregulated in involuting hemangioma samples (as it has been shown in the heatmap from Figure 3c). The second gene with divergent expression was the AKT1 gene, encoding RAC (Rho family)-alpha serine/threonine-protein kinase. This gene was upregulated in proliferating hemangioma and downregulated in involuting hemangioma (heatmap from Figure 3c). 

Mitogen-activated protein kinase 14 (MAPK14) was identified as having different expression in proliferating and involuting hemangiomas. It was found that MAPK14 was upregulated in proliferating hemangioma and showed downregulation in involuting hemangioma.

Ten out of twenty-sevens genes were detected to be upregulated for both types of hemangiomas: beta-actin (ACTB), Kirsten rat sarcoma virus gene (KRAS), dual specificity mitogen-activated protein kinase kinase 1(MAP2K1), Harvey rat sarcoma virus gene (HRAS), nitric oxide synthase 3 (NOS3), BCL2 associated agonist of cell death (BAD), heat shock protein beta-1 (HSPB1), hypoxanthine-guanine phosphoribosyl transferase (HPRT1), beta-glucuronidase (GUSB), and caspase-9 (CASP9). 

Thirteen genes were detected to be downregulated in both involuting and proliferating hemangiomas: C-fos-induced growth factor (FIGF), actin gamma 1 protein (ACTG1), Growth factor receptor-bound protein 2 (GRB2), MAP kinase-activated protein kinase 2 MAPKAPK2), actin gamma 2 protein (ACTG2), dual specificity mitogen-activated protein kinase kinase 2 (MAP2K2), mitogen-activated protein kinase 3 (MAPK3), heat shock protein subfamily AA (HSP90AA), dual specificity mitogen-activated protein kinase kinase 6 (MAP2K6), neuroblastoma Ras viral oncogene homolog (NRAS), actin-alpha skeletal muscle (ACTA1), kinase insert domain receptor (KDR), and mitogen-activated *protein* kinase 1 (MAPK1). A comparative assessment of VEGF pathway genes in involuting and proliferating hemangioma may be followed in Table 1.

## 4. Discussion

Involuting infantile hemangiomas are lesions which, after an intense proliferation, gradually involute spontaneously or following propranolol therapy use. However, after the involution, remaining lesions with a hemangioma morphology are still present in the involuted lesional area, as it also happened in our case, the infantile hemangioma being in the incipient involution phase. Recent literature has reported that involuting hemangiomas may reactivate under certain conditions such as the intravenous administration of therapeutic agents (e.g., salbutamol [14]) or after interrupting propranolol treatment.

Despite the serum and tissular VEGF overexpression certified for infantile hemangiomas [10,15,16], the intrinsic steps of the VEGF pathway gene expression profile are yet to be elucidated.

Experimental and clinical data show that the VEGF pathway is one of the most important pathways in the development of infantile hemangiomas [15,16,17]. Suppression of VEGF-A and VEGF-C induced a decrease in hemangioma cell growth by inhibiting cell proliferation and an enhancement in apoptosis [17]. Present discussions will be focused mainly on genes found to have divergent expression in proliferating and involuting hemangiomas in our study.

In our study, the AKT1 gene encoding RAC (Rho family)-alpha serine/threonine-protein kinase was upregulated in proliferating hemangioma. First evidence of AKT1’s role in the development of skin vascular malformations was reported by Perry et al. in 2007 [18]. They demonstrated in a mouse experimental model that sustained AKT1 activation and overexpression in MS1 endothelial cells injected subcutaneously induced vascular malformations with proliferating hemangiomas by stimulating endothelial cell proliferation independently of the Ras-mediated pathway [18]. Later, Phung et al. demonstrated that AKT1 upregulation promotes endothelial cell proliferation of human infantile hemangioma tissue, but their study did not clearly state whether the included human infantile hemangiomas were of the proliferating or involuting type. The pictures provided by the authors include human hemangioma tissue with proliferating hemangioma morphology and high AKT1 immunohistochemical expression. Our study clearly stated divergent AKT1 gene expression in between proliferating infantile hemangioma (upregulated) and involuting hemangioma (downregulated), supporting the previous studies’ findings related to AKT1 signaling as one of the main promoters of hemangioma endothelial cell proliferation and growth.

The past ISSVA classification mentioned the AKT1 gene as the causal agent of capillary, venous, and lymphatic malformations exclusively associated with Proteus Syndrome. Our patient had no history of Proteus Syndrome or any other genetic disease, but AKT1 was found to be upregulated in his proliferating hemangioma.

Propranolol therapy, which acts as a non-selective adrenergic receptor inhibitor, is still one of the most commonly used treatments for infantile hemangiomas. Several recent papers have reported that AKT1 expression is also influenced by propranolol therapy. Thus, AKT1 could be considered a therapeutic target for infantile hemangiomas and should receive more attention in recent times. 

A recent paper by Lee et al. reported that propranolol treatment inhibited migration, vascular network formation, vascular endothelial growth factor A production, and vascular endothelial growth factor receptor 2 activation, and downregulated the PI3K/AKT pathway but induced mitogen activated protein kinase pathway activation in hemangioma stem cells [19].

In our study, we detected an upregulation of MAPK14, a member of the MAPK family, in a case of proliferating hemangioma (not yet reported in the literature) together with AKT1 upregulation. MAPK14 was downregulated in involuting infantile hemangioma. MAPK14, also known as p38MAPK, is involved in the initiation of several disease states such as inflammatory disorders, neurodegenerative diseases, cardiovascular diseases, and cancer [4]. The role of MAPK14 in infantile hemangiomas is incompletely elucidated, with most of the data being derived from a few experimental studies [20]. The role of p38MAPK in hemangioma pathogenesis has been linked to the use of propranolol as a therapy [21,22,23].

Despite the long use of propranolol mainly as a therapy for proliferating hemangioma, its action and efficiency are still questionable. Mulliken discovered that propranolol acts differently on different cell types based on hemangioma structure using histochemical methods [24], and Glowacki observed hemangioma cell heterogeneity in hemangioma cells. Hemangioma-derived progenitor/stem cells (HemSCs), mesenchymal stem cells (Hem-MSCs), endothelial progenitor cells (HemEPCs), endothelial cells (HemECs), and perivascular cells (Hem-pericytes) have all been isolated from hemangioma tissues in recent decades [25,26,27,28]. HemSCs express markers for both HemEPCs and Hem-MSCs [29]. HemSCs implanted subcutaneously in nude mice are able to produce GLUT1-positive small blood vessels in about 2 weeks after implantation [30,31,32]. Both involuting and proliferating hemangiomas from the present study expressed GLUT1.

Induced inhibition of hemangioma endothelial cell proliferation and migration by inhibiting AKT1 and p38MAPK pathways [21] in a dose dependent manner is unable to suppress clonogenic GLUT1-positive HemSCs, which have the ability to continuously differentiate into other proliferating hemangioma endothelial cells [33]. This is most likely one of the mechanisms favoring recurrences after propranolol. Thus, rapamycin therapy seems to be more effective than propranolol alone [34] for some proliferating hemangiomas.

There was no previous research on the interaction of p38MAPK with HemSCs. Batlle et al. demonstrated in mice that p38MAPK signalling modulates the differentiation of mesenchymal stem cells through an endothelial phenotype [35]. The same authors reported that inhibition of p38MAPK stimulated mesenchymal to endothelial transition followed by endothelial cell differentiation and activation. Based on the literature and our findings, we can speculate that propranolol inhibits p38MAPK, which inhibits HemEC proliferation and migration in infantile hemangiomas. On the other hand, p38MAPK inhibition may recruit HemSCs and potentiate them to differentiate into other active HemECs. In this way, MAPK14 may play a dual role in infantile hemangioma pathogenesis and may potentiate the incomplete response and recurrences that sometimes follow after propranolol treatment in proliferating infantile hemangiomas.

The versatile role of p38/MAPK14 is reported to also be related to perivascular cell acquisition [35]. p38/MAPK14 downregulation is followed by an increased acquisition of perivascular cells responsible for vessel maturation and stabilization. In our study, involuting infantile hemangioma was characterized by downregulation of p38/MAPK14 and upregulation of the ACTA2 gene. The ACTA2 gene is known as a promoter of perivascular cell recruitment and development, being also expressed by fibroblasts and myofibroblasts [36,37]. Its role is mainly related to fibrosis in several organs such as the kidney, lung, heart, liver, skin, and bone marrow [36]. Immunohistochemical overexpression of smooth muscle actin (encoded by the ACTA2 gene) in hemangioma cells, mainly in sclerosing lesions, has been very rarely reported in the literature in human tissues. The only two papers in the literature that reported smooth muscle actin immunohistochemical overexpression in sclerosed zones of hepatic hemangiomas are those of Makhlouf and Ishak in 2002 [37] and Shimada et al. [38].

Experimental data about propranolol’s effects on involution of hemangioma related to its effects on smooth muscle actin expressing cells are also very rare. Lee et al. showed that hemangioma-derived pericytes express high levels of _2_ AR mRNA and that this receptor is relatively high in infantile hemangiomas. Treatment with hemangioma-derived pericytes resulted in increased pericyte contractility [4] as well as a decrease in blood flow to hemangioma capillaries.

No data about ACTA2 gene upregulation nor its association with involuting hemangioma has been reported before the present study. Previous published data, combined with ACTA2 upregulation in involuting hemangioma, suggest that hemangioma pericytes play an important role in hemangioma involution via at least two possible mechanisms: (1) increased number and contractility of hemangioma-derived pericytes influencing capillary maturation and perfusion, and (2) the ability of hemangioma pericytes to transdifferentiate into myofibroblasts favoring HEMA. Preliminary data support ACTA2 upregulation as one of the mechanisms of hemangioma involution, but more research is needed to determine its role in hemangioma regression.

The main weakness of the present study may be that it was performed by comparing only two cases of proliferating and involuting hemangiomas, but it is the first report highlighting the divergent gene expression of AKT1, MAPK14, and ACTA2, and is not previously reported in involuting and proliferating infantile hemangiomas.

Previous published papers in the field presented scattered observations from case presentations mainly or experimental models. All the previously presented data suggest: (1) the need for extensive studies on the large cohort of hemangioma cases and (2) the inclusion of an extended gene expression profile into future classification of vascular malformations with an emphasis on proliferating and infantile hemangiomas.

Most of other MAPK family members were downregulated for both involuting and proliferating hemangioma except MAP2K1 which was found to be upregulated for both types of our hemangioma. Compared with the previously discussed upregulated genes (AKT1 and MAPK14) which have not yet been reported as having an involvement in hemangioma pathogenesis, MAP2K1 is highly present in several papers focused on vascular malformations [39,40,41] as a gene having somatic mutation associated with vascular malformation. Most of the papers reporting MAP2K1 somatic mutation frequently associated KRAS mutation [39,40,41] and HRAS mutation [42]. We also found an upregulation of KRAS and HRAS genes for both involuting and proliferating hemangioma. A somatic activating NRAS mutation was detected in kaposiform lymphangiomatosis but not in kaposiform hemangioendothelioma [43]. NRAS was the only member of the RAS family included in the present study which was downregulated in both infantile and proliferating hemangioma. Except for the previous mentioned article [43], no other studies on infantile hemangiomas or other vascular malformations reporting NRAS gene involvement have been published.

The assessment of the VEGF pathway gene expression profile in hemangiomas may have a clinical impact through its use for patients’ stratification related to hemangioma progression and their possible recurrences.

The clinical impact of gene expression profiles in hemangiomas is not well defined because all information is reported on a small cohort of cases or even related to scattered case presentations published in the literature. Based on gene expression profiles, Walter et al. [44] identified three gene candidates: fibroblast growth factor receptor-4 (FGFR4), platelet-derived growth factor receptor-beta (PDGFR-B), and fms-related tyrosine kinase-4 (FLT4), which characterize familial hemangiomas. All these genes are also involved in angiogenesis, which is closely related to the VEGF pathway. Grimmer et al. [45] assessed the relative risk for people with familiar hemangiomas in 2011 and found that it is 2-fold higher for siblings.

Validation of serum biomarkers predicting therapy response and recurrences must be sustained by an initial tissular biomarker evaluation and gene expression profile assessment related to the VEGF pathway for hemangiomas tissues. Identification of genes responsible for recurrences should become mandatory before therapy. The strongest impact of gene expression profile analysis seems to be on therapy selection and therapy response in different types of hemangiomas. Intralesional injection of bevacizumab (anti-VEGF therapy) has already been applied for retinal [46], choroidal [47], and sinonasal hemangiomas [48] with promising results. An accurate gene expression profile assessment followed by patient stratification will drive the selection of the most targeted therapies. AKT1 is a therapeutic target for AKT1 inhibitors, which have been shown to be effective in inhibiting breast cancer cell lines [49]. Phung et al. reported AKT1 signaling inhibition by rapamycin in endothelial cells from tumor blood vessels followed by vessel normalisation [50]. Recently, Davila-Osorio et al. reported the successful treatment of one propranolol-resistant infantile hemangioma with sirolimus [51]. There are no data about p38/MAPK14 inhibitors’ use in clinical practice as one potential targeted therapy for proliferating infantile hemangiomas.

## 5. Conclusions

AKT1, p38/MAPK14 (upregulated in proliferating hemangioma but downregulated in involuting hemangioma), and ACTA2 (upregulated in involuting hemangioma but downregulated in proliferating hemangioma) were found to have divergent expression in proliferating and involuting hemangiomas. Except for AKT1, which was mentioned in the last ISSVA classification (strictly related to Proteus Syndrome), none of the mentioned genes were reported as having an impact on infantile hemangioma development or their therapy response. Previous scattered published data and the current study’s findings encourage the development of large studies in the future to validate gene expression profile roles in hemangioma progression and therapy response. An accurate gene expression profile mapping of infantile hemangiomas together with gene expression-based hemangioma classification is stringently needed.

## Figures and Tables

**Table 1:** Gene amplification differences between the involuting and proliferative hemangioma.

Gene	Involuting Hemangioma	Proliferative Hemangioma
ACTB	Upregulate	Upregulate
FIGF	Downregulate	Downregulate
KRAS	Upregulate	Upregulate
MAPK14	Downregulate	Upregulate
PiK3CA	Not expressed	Not expressed
ACTG1	Downregulate	Downregulate
GRB2	Downregulate	Downregulate
MAP2K1	Upregulate	Upregulate
MAPKAPK2	Downregulate	Downregulate
ACTG2	Downregulate	Downregulate
HRAS	Upregulate	Upregulate
MAP2K2	Downregulate	Downregulate
MAPK3	Downregulate	Downregulate
HSP90AA1	Downregulate	Downregulate
NOS3	Upregulate	Upregulate
BAD	Upregulate	Upregulate
HSPP1	Upregulate	Upregulate
MAP2K6	Downregulate	Downregulate
NRAS	Downregulate	Downregulate
AKT1	Downregulate	Upregulate
ACTA1	Downregulate	Downregulate
ACTA2	Upregulate	Downregulate
CASP9	Upregulate	Upregulate
KDR	Downregulate	Downregulate
MAPK1	Downregulate	Downregulate
